# A 3D-printed patient-specific modular implants for pelvic reconstruction of bone tumors involving the sacroiliac joint

**DOI:** 10.3389/fbioe.2023.1233960

**Published:** 2023-08-24

**Authors:** Zhaorui Lv, Zhenfeng Li, Qiang Yang, Jianmin Li

**Affiliations:** Department of Orthopedics, Qilu Hospital, Shandong University, Jinan, Shandong, China

**Keywords:** sacroiliac joint, reconstruction, tumor, 3D-printed, patient-specific, implant

## Abstract

**Background:** Current reconstruction methods of the pelvic ring after extensive resection of tumors involving the sacroiliac joint have a high incidence of failure. We aimed to study the effect of 3D-printed patient-specific implant reconstruction to show that this method is stable and has a low risk of failure.

**Methods:** Between February 2017 and November 2021, six patients with bone tumors involving the sacroiliac joint (Enneking I + IV) who received 3D-printed patient-specific implants for pelvic reconstructive surgery were retrospectively analyzed. Two female and four male patients with a mean age of 41.83 years (range 25–65 years) were included. Two were osteosarcomas, two chondrosarcomas, one malignant fibrous histiocytoma, and one giant cell tumor of bone. For each patient, preoperative osteotomy guides were designed to ensure accurate tumor resection and individualized prostheses were designed to ensure a perfect fit of the bone defect. General, oncologic, and functional outcomes, implant status, and complications were retrospectively analyzed. The Visual Analog Scale (VAS) was used to assess pain and the Musculoskeletal Tumor Society (MSTS) score was used to assess hip function. Osseointegration was assessed by CT.

**Results:** According to the preoperative design, complete resection of the entire tumor and reconstruction with a custom 3D-printed sacroiliac joint implant was completed without perioperative severe complications or deaths. Relatively satisfactory surgical margins were achieved. The mean operative time and intraoperative blood loss were 495 min (420–600 min) and 2533.33 mL (range, 1,200–3,500 mL), respectively. The mean follow-up was 49.83 months (range, 18–75 months). At the last follow-up, all four patients were disease-free, and the two patients who developed lung metastases were alive with tumors. All patients could walk unassisted. The mean VAS was 1.33 (range, 0–2). The mean MSTS score was 25.33 (range, 24–27). CT showed complete osseointegration of the implant to the ilium and sacrum.

**Conclusion:** The 3D-printed custom prosthesis can effectively reconstruct pelvic stability after total sacroiliac joint resection with satisfactory clinical results.

## Introduction

The pelvis is a common site for bone tumors (Bloem and Reidsma, 2012). Limb salvage surgery has gradually replaced amputation as the standard of care for pelvic tumors over the years as surgical techniques have evolved (Puri et al., 2014). The complex anatomy of pelvic tumors presents a significant challenge to orthopedic oncologic surgeons. Enneking type I + IV resection is used to remove tumors that invade the sacroiliac joint, which disrupts the pelvic ring (Zoccali et al., 2023).

Mobility, pain, and quality of life can be improved by reconstructing the anatomical pelvic ring. Many reconstruction methods have been proposed: Autograft, allograft, or implant (Zang et al., 2014; Ogura et al., 2015; Zhou et al., 2016). Each method has its pros and cons. Current reconstructive procedures have a high rate of complications (Sabourin et al., 2009; Zang et al., 2014). These include implant failure, wound complications, and surgical site infections. In order to ensure adequate function and quality of life after surgery, much effort is needed to improve patient survival.

The use of 3D printing in orthopedic surgery now makes it possible to accurately design implants for complex bone defects based on biomechanical factors (Barbera et al., 2019; Pu et al., 2021; Danielli et al., 2023). Biomechanical factors include satisfactory mechanical properties and structural stability (Li et al., 2020). This allows for efficient evaluation and creation of new, personalized designs to meet clinical needs. In certain cases, the use of 3D-printed patient-specific implants is on the rise for the reconstruction of severe tumor bone defects (Kotrych et al., 2023). 3D-printed prostheses offer the advantage of a precise fit and good osseointegration (Lee et al., 2022).The use of 3D-printed implants for sacral tumor resection reconstruction is safe, effective, and contributes to better functional outcomes, as we have previously reported (Lv et al., 2020; Lv et al., 2023a; Lv et al., 2023b; Lv et al., 2023c). We hypothesize that 3D-printed patient-specific implants may reduce implant failure rates.

Therefore, we established new 3D-printed patient-specific implants. The design concept is an implant with a bioactive porous interface that connects the residual sacrum to the residual iliac bone in one step. The prosthesis we designed is modular and has two components. This design allows for easy fitting and revision. Reliable data on reconstruction with this prosthesis are lacking due to the rarity of these cases. This study aimed to evaluate the long-term clinical outcome and complications of 3D-printed prostheses based on 6 consecutive patients.

## Materials and methods

### Patients

**FIGURE 1:**
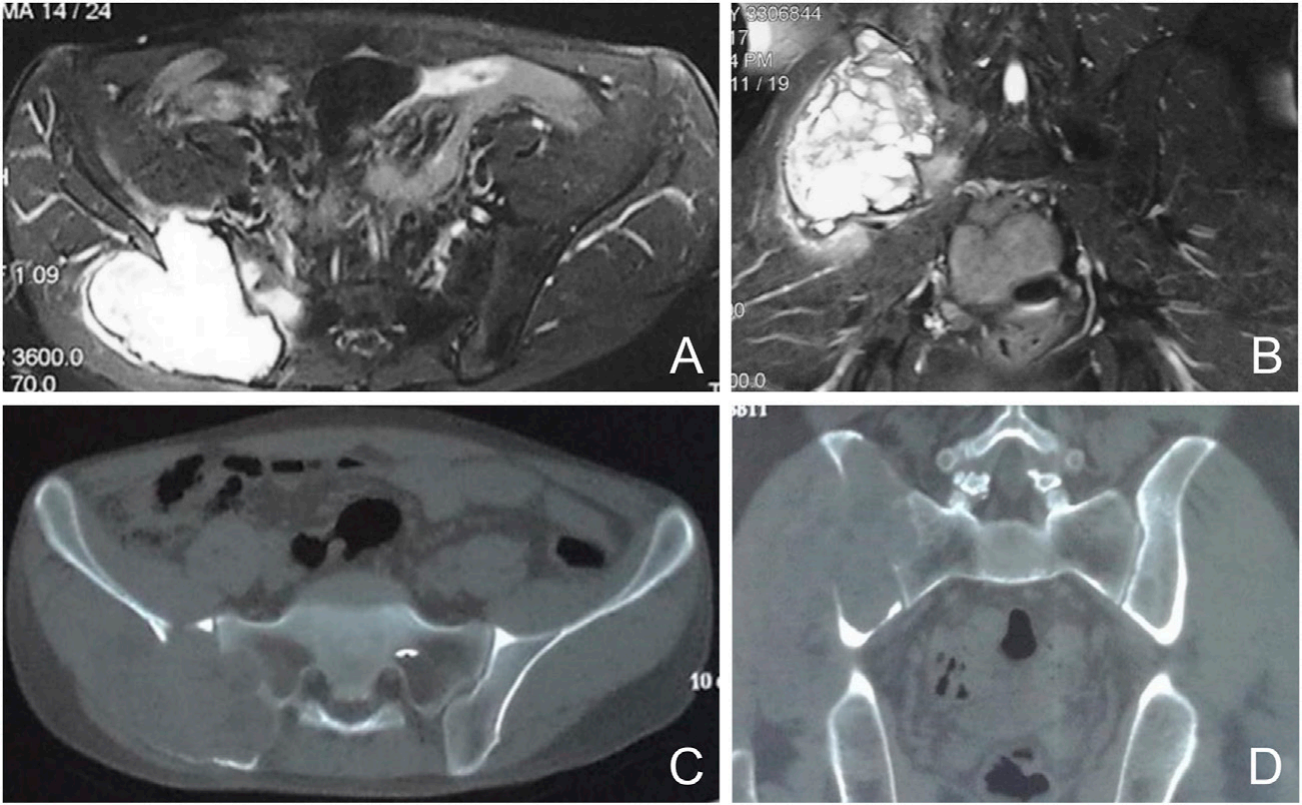
Preoperative imaging of a representative case. Axial and coronal nuclear magnetic resonance images show a cumulative lesion of the sacroiliac joint with edema **(A, B)**. Axial and coronal computed tomography images show a cumulative osteolytic lesion of the sacroiliac joint **(C, D)**.

From February 2017 to November 2021, we retrospectively analyzed six patients with sacroiliac joint tumors (Enneking and Dunham zones I and IV) who underwent 3D-printed personalized implants for pelvic reconstructive surgery at our institution. Five of them were male and one female, aged 25–65 years, with a mean age of 41.83 years. Two cases of chondrosarcoma, two cases of osteosarcoma, one case of malignant fibrous histiocytoma, and one case of giant cell tumor of bone were found. Exclusion criteria included reconstruction with systems other than custom-made prostheses (e.g., screws, rods, large allografts, autologous fibula, etc.). Thus, all patients presented with pain and no fracture at the time of diagnosis. The average time of onset of symptoms was 4 months before diagnosis (range 3–6 months). Plain radiographs, CT and pelvic magnetic resonance imaging (MRI) were performed (Figure 1). Patients underwent CT-guided puncture biopsy and histopathologic diagnosis was performed by a pathologist. Patient medical records, imaging, oncology, and functional status were reviewed.

### 3D-printed implant design and fabrication

**FIGURE 2:**
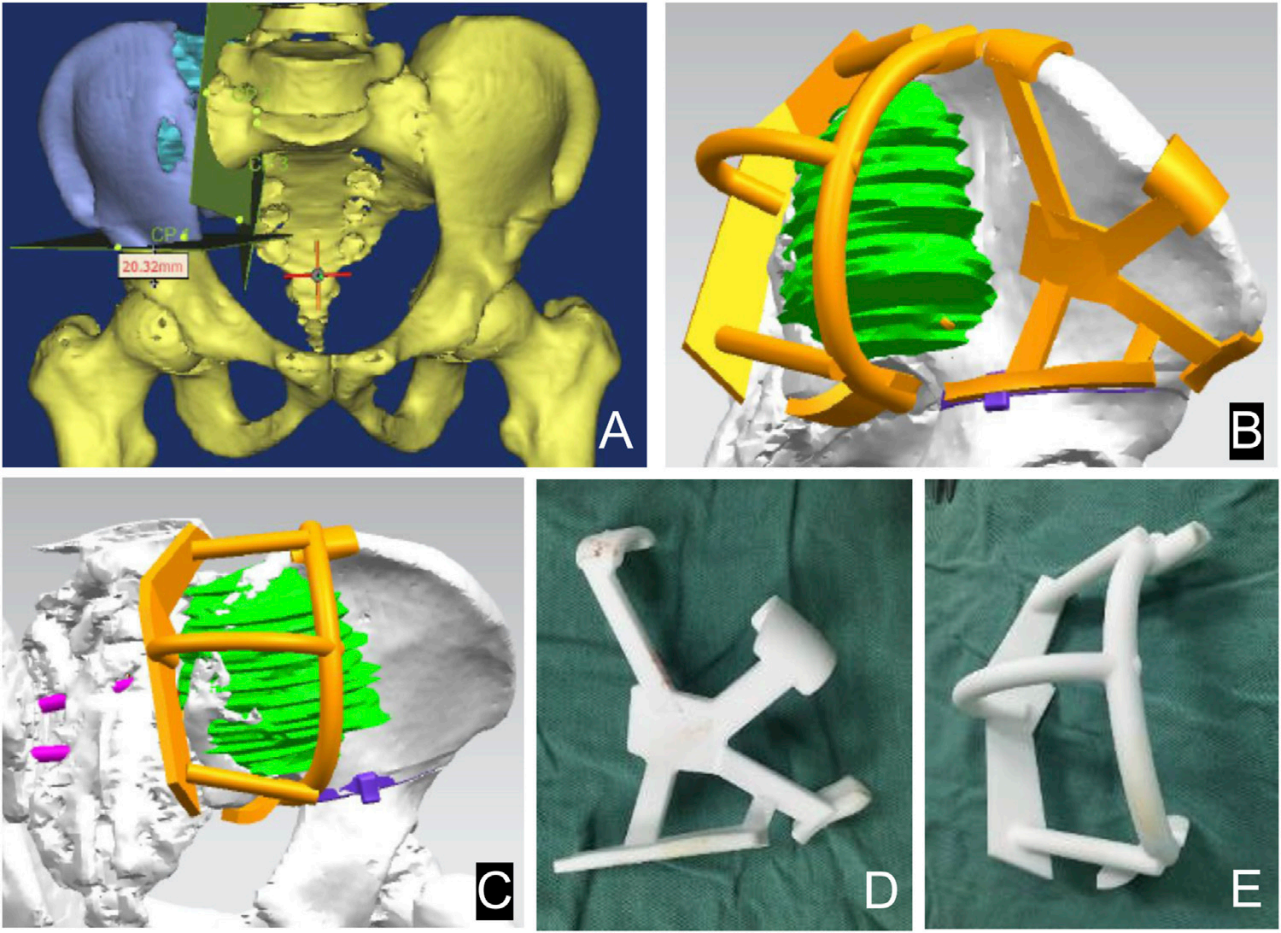
Design of the 3D bone tumor model and cutting guide. The 3D bone tumor model created from CT data was used for surgical planning **(A)**. The personalized cutting guide designed by computer **(B, C)**. External view of the cutting guide manufactured by 3D printing technology **(D, E)**.

**FIGURE 3:**
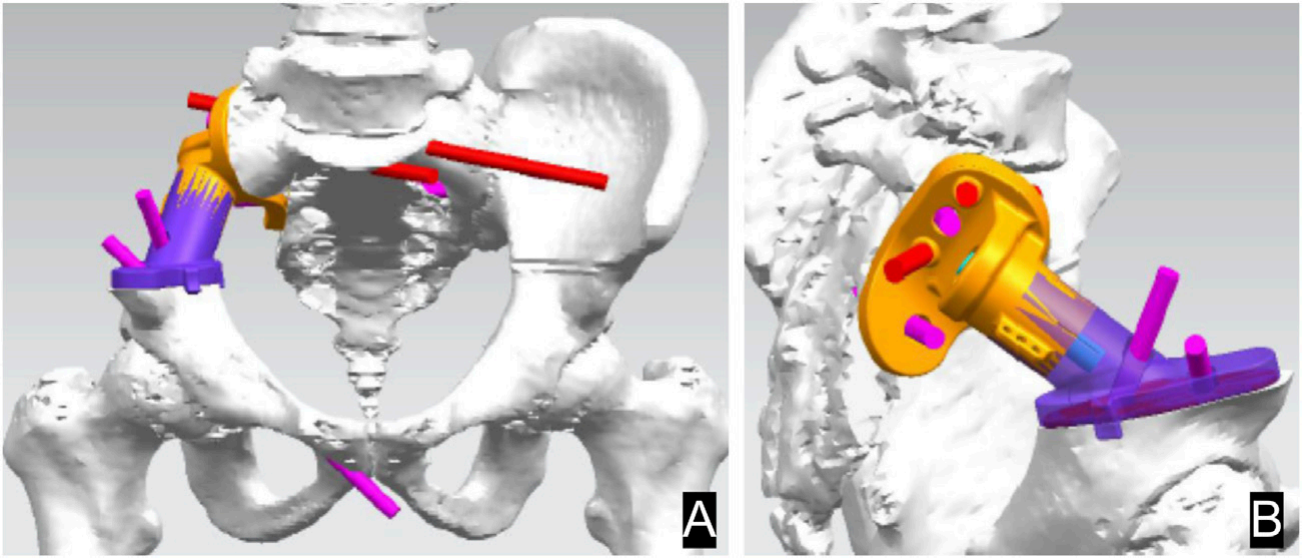
Design of the sacral implant. Front view **(A)** and side view **(B)** of the implant.

The 3D prosthesis and patient-specific instruments are designed by our team. The patient’s CT data are stored in DICOM format. The data were imported into Materialize Mimics V17.0 software (Materialize Corp., Leuven, Belgium) to create virtual 3D tumor and pelvic bone models. Safe tumor cutting edges were determined on the model (Figure 2). Based on the desired cutting plane, patient-specific surgical instruments (PSI) were designed to provide accurate guidance during intraoperative osteotomy (Figure 2). Each PSI has a unique fixation device designed to follow the skeletal morphology of the specific patient (Figure 2). Prosthesis design was performed using Geomagic Studio software (Geomagic Inc., Morrisville, United States) (Figure 3). The design of the integrated prosthesis was adapted to the resected bone defect. Multiple screw channels were present at the junction of the lateral sacrum and the top of the acetabulum. Unnecessary features were removed and the surface of the prosthesis was smoothed. The prosthesis was fabricated with a metal 3D printer system (EOS M290, EOS GmbH Electro Optical Systems, Munich, Germany) using medical-grade Ti-6Al-4V powder.

### Surgical techniques

**FIGURE 4:**
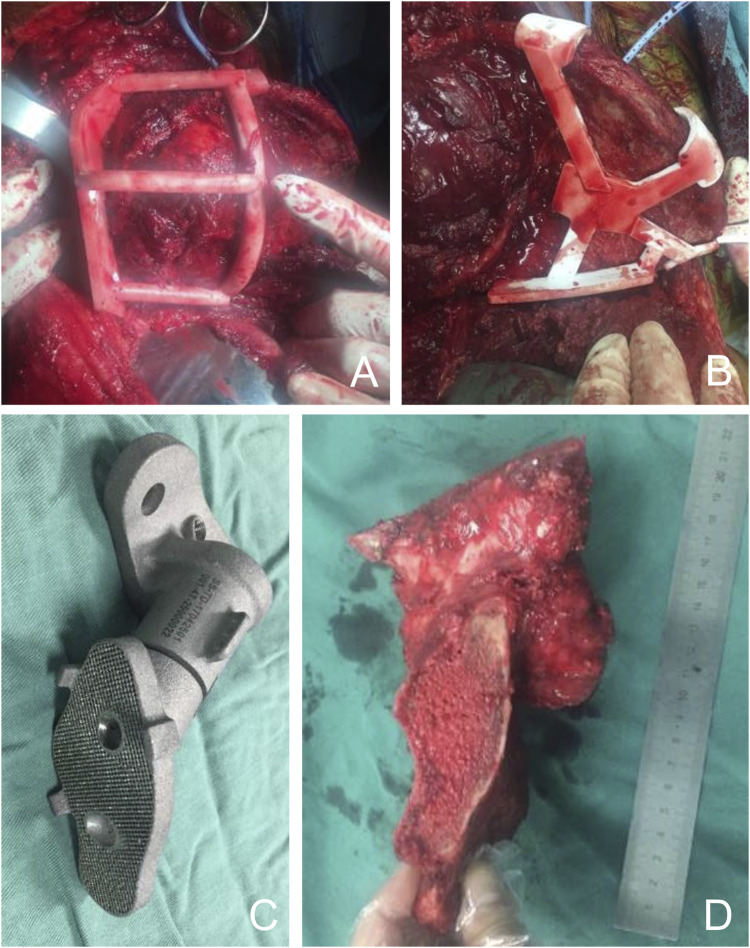
Intraoperative images. **(A, B)** Using the cutting guide, the iliac bone and sacrum were precisely dissected and the tumor was resected as planned preoperatively. **(C)** The 3D printed implants were sterilized and sent to the surgeon for backup. **(D)** The tumor was completely removed and the surgical margin was ideal.

To reduce intraoperative bleeding, all patients underwent preoperative selective arterial embolization and intraoperative aortic balloon occlusion. The procedure is performed with the patient in the lateral decubitus position. An extended iliac crest approach is used. In the medial pelvis, the abdominal muscles are separated from the iliac crest to expose and protect the external iliac and femoral vessels and nerves. Dissection is performed medial to the pelvis to the anterior sacrum. Lateral to the pelvis, the gluteal muscles are separated, preserving the gluteal neurovascular structures and exposing the external iliac crest to the posterior aspect of the sacrum. The PSI was fixed with multiple 2-mm Kirschner pins and osteotomized around the sacroiliac joint with an ultrasonic osteotome. The tumor was excised intact as planned (Figure 4). A 3D-printed personalized implant was placed in the bone defect for reconstruction (Figure 4). To fix the prosthesis, multiple cancellous screws were inserted through holes in the top of the prosthesis into the S1 or S2 vertebrae, and two additional screws were inserted through the top of the acetabulum into the pubic bone and sciatic bone, respectively. Reconstruction was performed according to the preoperative plan. All unresected muscles were sutured and fixed to the prosthesis. Two drains were placed and the wound was closed in layers. The resection specimens were analyzed by a professional pathologist.

### Postoperative treatment and follow up

The patient was encouraged to begin early rehabilitation on the first postoperative day. Patients began active hip flexion and extension at 2 weeks. Partial weight bearing was initiated at 4 weeks, followed by full weight bearing as tolerated. All malignancies and patients received adjuvant chemotherapy regimens after incisional healing. Follow-up visits were every 3 months for the first year, every 6 months for the second year, and annually thereafter. Physical examination and imaging were performed at each visit. Survival was assessed by local and distant tumor control. Chest computed tomography (CT) was performed to detect pulmonary metastases.

Osseointegration at the bone-prosthesis interface was assessed by CT and radiography of the surgical site to detect possible local recurrence. There are three types of osseointegration: 1) Complete osseointegration: continuous bone trabeculae throughout the bone-prosthesis interface; 2) partial osseointegration: some continuous and orderly bone trabeculae running through part of the area, and part of the area without osseointegration as small or scattered hypodense areas. 3) No osseointegration: the bone-prosthesis interface shows a low-density area of bone resorption surrounded by a circular sclerotic zone, often accompanied by prosthesis loosening, displacement, and fracture. Functional outcomes were assessed using the Musculoskeletal Tumor Society (MSTS) scores (Enneking et al., 1993). Clinical complications including infection, incisional complications, and loosening were recorded.

### Statistical analysis

Descriptive statistics were used to summarize demographic characteristics and outcomes. For descriptive statistics, values are reported as mean and range.

## Results

### Operational outcomes

According to the preoperative design, complete resection of the entire tumor and reconstruction with a custom 3D-printed sacroiliac joint prosthesis were completed. Relatively satisfactory surgical margins were achieved and local tumor recurrence was minimized (Table 1). The mean operative time and intraoperative blood loss were 495 min (range, 420–600 min) and 2533.33 mL (range, 1,200–3,500 mL), respectively. Pathology results showed negative margins in all patients.

**TABLE 1:** Summary data for all patients.

No	Age	Sex	Pathologic diagnosis	Blood loss (ml)	Operating time (min)	Follow-up (months)	MSTS score	VAS score	Complications	Time to complete osseointegration (months)	Survival status
1	65	M	Chondrosarcoma	3,000	600	75	25	2	delayed wound healing	3	no evidence of disease
2	38	M	giant cell tumor of bone	1,200	420	72	27	0	None	3	no evidence of disease
3	31	F	Malignant fifibrous histiocytoma	3,000	540	58	25	2	delayed wound healing	6	Alive with disease
4	57	M	Chondrosarcoma	3,500	480	42	26	2	None	6	no evidence of disease
5	35	M	Osteosarcoma	2,000	420	34	25	1	None	3	Alive with disease
6	25	M	Osteosarcoma	2,500	510	18	24	1	None	3	no evidence of disease

### Oncologic outcomes

At the last follow-up, all four patients were disease-free, and the two patients who developed lung metastases were alive with tumors and without local recurrence.

### Functional outcome

At 3 months postoperatively, all patients could walk unassisted and had no gait disturbances. The mean VAS was 1.33 (range, 0–2). The mean MSTS score at the last follow-up visit was 25.33 (range, 24–27).

### Complications

No serious complications or deaths occurred during the perioperative period. Wound healing was delayed in two patients. These wound problems were successfully resolved by timely debridement, drainage, and antibiotics. No deep infections occurred. The mean follow-up was 49.83 months (range 18–75 months).

### Implant status

**FIGURE 5:**
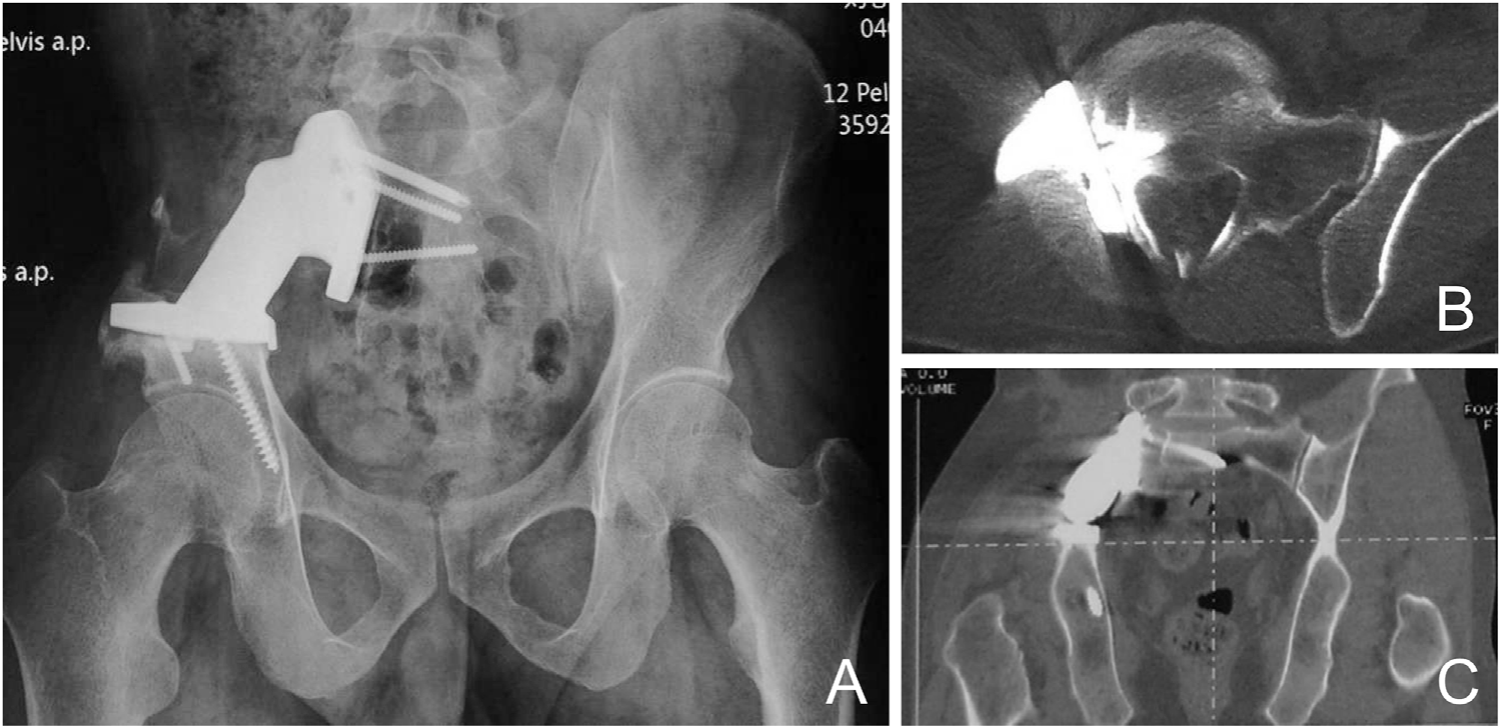
Prosthetic Osseointegration **(A)** X-rays show a well positioned prosthesis with no use of loose displacement or fracture. CT shows good osseointegration of the implant with the adjacent bone in the sagittal **(B)** and coronal planes **(C)**.

Imaging data showed that the 3D-printed prosthetic device was well positioned with no internal fixation failure in the form of loosening, displacement, or breakage (Figure 5).CT showed full osseointegration of the prosthetic device with the adjacent bone (Figure 5). We consider the absence of gaps at the bone-prosthesis interface with the presence of continuous trabeculae as good osseointegration. The mean time to osseointegration was 4 months (range 3–6 months). Patients with good osseointegration did not experience displacement or screw loosening.

## Discussion

With digestive, urinary, and reproductive organs, as well as important blood vessels and nerves, the anatomy of the adjacent pelvic area is complex. Pelvic tumors are difficult to resect precisely, and pelvic defects after resection are complex and vary greatly from individual to individual. In the past few years, the development of medical 3D printing technology has provided new ideas for the precise resection and personalized reconstruction of the pelvic tumor. 3D-printed pelvic tumor models can assist the surgeon in the removal and reconstruction of the tumor before surgery. To reduce the risk and time of surgery, 3D-printed pelvic tumor models can help surgeons plan and simulate surgery before surgery. During the intraoperative process, 3D-printed osteotomy guides can assist the surgeon. 3D-printed personalized reconstruction prosthesis conforms to pelvic defect shape, enabling precise reconstruction after pelvic tumor removal.

In this study, six consecutive patients underwent 3D-printed personalized implant reconstruction after Enneking type I + IV primary bone tumor resection (Otsuki et al., 2021). To improve the stability of the reconstruction and allow for an early return to function, the prosthesis is screwed to the sacrum and acetabular roof. The 3D-printed pelvic construct in this study was easy to assemble due to its highly conformable contour shape and predetermined nail trajectories and fixation points, allowing for rapid positioning and precise fixation of the screws in the construct after intraoperative osteotomy. Pelvic prosthesis reconstruction has the advantages of ease of insertion, immediate stability, early rehabilitation, and avoidance of complications such as secondary deformity and long-term fracture. In addition, more stable fixation and closer to normal mechanical conduction can be achieved by using long cancellous screws in combination with short cortical nails for prosthetic fixation. MSTS scores were satisfactory at the final follow-up.

Determining the correct resection margins is an important key to achieving extensive resection and reducing the risk of local tumor recurrence. The 3D-printed Pelvic Osteotomy Guide is a personalized, computer-aided design, a 3D-printed surgical device used for intraoperative positioning and to assist in the precise creation of the osteotomy. The 3D-printed pelvic osteotomy guide can assist the surgeon in effectively determining the plane and direction of the osteotomy, simplify the operation, reduce the number of intraoperative fluoroscopies, shorten the operation time, improve the accuracy of tumor resection, and effectively reduce tumor recurrence (Gasparro et al., 2022). In our study, we did not observe any local recurrence during the 4-year follow-up, probably because the use of 3D cutting guides in the resection ensured the correct resection margins and a precise resection was achieved.

In recent years, solutions for reconstructing difficult sites such as the sacroiliac joint have been provided by the development of personalized implants (Yu et al., 2021). In recent years, reconstruction with perfectly matched implants in large bone defects after tumor resection has become possible through the application of computer-aided design and 3D printing technologies (Zoltan et al., 2023). The 3D Printing Pelvic Reconstruction Device is a customized surgical device designed to fit the pelvis defect. The 3D printing pelvic reconstruction also considers the repair of the pelvic defect and the integrity of the pelvic ring, including the sacroiliac joint and the symphysis pubis, as well as the restoration of hip function. To achieve initial and long-term stability after reconstruction, the anatomical, mechanical, and biological adaptations of the reconstructed prosthesis are optimized. The goal is to achieve anatomical reconstruction by perfectly matching the implant and the host bone to achieve better function and reduce the complication rate, thus improving the functional outcome (Wang et al., 2020). In oncologic surgery, many authors consider the mechanical complication rate of these implants to be lower than that of other reconstructions (Pu et al., 2021).

3D printed porous implants are biocompatible to promote osseointegration. They are fully capable of handling complex mechanical environments (Shen et al., 2022). Osseointegration between the residual pelvis and the prosthesis is important to prevent implant failure (Liang et al., 2017). The prosthesis has the unique advantage of long-term stability with a low rate of internal fixation failure and no patients have experienced prosthesis loosening or nail fracture. Poor osseointegration can be a result of a small contact area, reduced blood flow, stress shielding of the implant, etc. Indications for the use of a 3D printed custom pelvic prosthesis include: 1) The patient’s systemic and local conditions allow for surgery; 2) the pelvic bone defect needs to be repaired for any reason; 3) the conventional prosthesis cannot meet the requirements of repairing this bone defect or the repair is difficult; 4) it is consistent with the principles of tumor surgical treatment. Contraindications include: 1) The patient’s systemic and local conditions contraindicate surgery; 2) the bone defect can be repaired with a conventional prosthesis or otherwise; 3) patients and their families do not want to use it or cannot afford the medical expenses involved.

Several limitations must be addressed. This is a consecutive retrospective series dealing with the management of a rare clinical condition. The number of patients was small. Statistical analysis could not be performed due to the limited sample size. The incidence of mechanical complications such as aseptic loosening and screw fracture may be underestimated due to the short follow-up period. Longer follow-up is needed to confirm the efficacy of the 3D-printed pelvis for sacroiliac joint stabilization. Another limitation is that this is a retrospective study, which is subject to selection bias. The efficacy of the 3D-printed pelvis for sacroiliac joint stabilization will be confirmed with longer follow-up measurements. Finally, the effect of the 3D-printed material on bone ingrowth needs to be monitored over time.

## Conclusion

The 3D-printed custom prosthesis can effectively restore the continuity and stability of the pelvis after tumor resection of the sacroiliac joint with satisfactory clinical results, good osseointegration, and durability, which is worthy of clinical promotion. Our reconstruction method allows early rehabilitation, preserves ambulation, and the perioperative complication rate is not high. Further followup assessment in a larger study population is required in these patients.

## Data Availability

The original contributions presented in the study are included in the article/Supplementary Material, further inquiries can be directed to the corresponding author.
